# Les mucocèles appendiculaires, problèmes diagnostiques et thérapeutiques: à propos de 26 cas

**DOI:** 10.11604/pamj.2015.22.362.8354

**Published:** 2015-12-14

**Authors:** Mohamed Hedfi, Med Taieb Jomni, Dorra Ben Ghachem, Cherif Abdelhedi, Adnene Chouchene

**Affiliations:** 1 Hôpital des FSI la Marsa, Rue Fadhel Ben Achour 2070, Tunisie

**Keywords:** Mucocèle appendiculaire, chirurgie, cystadénocarcinome, appendix mucocele, surgey, cystadenocarcinoma

## Abstract

La mucocèle appendiculaire est une affection rare caractérisée par une dilatation de la lumière appendiculaire avec des altérations muqueuses linéaires, une hypersécrétion de mucus. Nous avons réalisé une étude rétrospective entre le mois de janvier 2000 et le mois Décembre 2014. Vingt six patients dont 16 hommes et 10 femmes ayant un âge moyen de 43 ans ont été colligés. Les douleurs abdominales étaient un symptôme constant. Quatre malades (15%) avaient une masse palpable au niveau de la fosse iliaque droite et un autre avait une ascite lors du diagnostic. Une complication à type d'occlusion intestinale aigue était révélatrice dans deux cas. Le diagnostic était évoqué en préopératoire par l'imagerie chez neuf (34%) patients. Une appendicectomie simple était réalisée chez 8 patients. Trois malades ont eu une résection iléo-caecale devant un aspect per opératoire de masse appendiculaire. Le geste était une hémi colectomie droite avec anastomose iléo transverse pour 5 patients, une résection iléo-caecale pour deux patients et une réduction tumorale associée à une évacuation de l'ascite dans un autre cas. L'examen anatomo-pathologique avait conclu à une mucocèle rétentionnelle bénigne, un cystadénome mucineux et à un cystadénocarcinome chez respectivement 15, 7 et 4 malades.

## Introduction

La mucocèle appendiculaire (MA) est une affection rare représentant seulement 0.3% des pièces d'appendicectomies. Elle est caractérisée par une dilatation de la lumière appendiculaire avec des altérations muqueuses linéaires, une hypersécrétion de mucus et occasionnellement un épanchement muqueux intrapéritonéal (EMI) ou plus rarement des lésions secondaires à distance. En se basant sur une étude rétrospective colligeant 26 cas de tumeurs mucosécrétantes de l'appendice; nous nous proposons de revoir la physiopathologie, les caractéristiques cliniques, anatomopathologiques, ainsi que les modalités diagnostiques et thérapeutiques de ces tumeurs

## Méthodes

Il s'agit d'une étude rétrospective réalisée au centre hospitalier des FSI la Marsa entre le mois de janvier 2000 et le mois Décembre 2014. Tous les patients ayant une mucocèle appendiculaire confirmée par un examen anatomopathologique ont été colligés. Leurs caractéristiques épidémiologiques, cliniques, morphologiques et les modalités de leurs prises en charge thérapeutique ont été analysées.

## Résultats

Vingt six patients ont été colligés dans l’étude. Une prédominance masculine était noté (16 hommes et 10 femmes) et l’âge moyen était de 43 ans avec des extrêmes de 17 à 87 ans. Les circonstances de découverte étaient dominées par les douleurs abdominales de la fosse iliaque droite (FID) qui était un symptôme constant chez tous nos patients. Quatre malades (15%) avaient une masse palpable au niveau de la FID et un autre avait une ascite lors du diagnostic. Une complication à type d'occlusion intestinale aigue était révélatrice dans deux cas. Le diagnostic de mucocèle était évoqué en préopératoire par une échographie et ou un scanner chez neuf (34%) patients. La coloscopie réalisée chez deux patients avait objectivé dans les 2 cas la présence du mucus au niveau de l'orifice appendiculaire. Tous les malades ont eu un traitement chirurgical. Parmi eux dix huit étaient opérés en urgence devant la suspicion en préopératoire d'une appendicite aigue. Une appendicectomie simple sous coelioscopie et sous incision de Mac Burny était réalisée chez respectivement 6 et 12 patients. Trois malades ont eu une résection iléo-caecale devant un aspect per opératoire de masse appendiculaire. Huit patients étaient opérés à froid par voie médiane. Le geste était une hémi colectomie droite avec anastomose iléo transverse pour 5 patients, une résection iléo-caecale pour deux patients et une réduction tumorale associée à une évacuation de l'ascite dans un autre cas. L'examen anatomo-pathologique avait conclu à une mucocèle retentionnelle bénigne, un cystadénome mucineux et à un cystadénocarcinome chez respectivement 15, 7 et 4 malades.

## Discussion

Les MA sont rares (0.2 à 0.3% des pièces d appendicectomies) et fréquemment rapportées chez les sujets de sexe féminin de plus de 50 ans contrairement à notre série ou il y avait une prédominance masculine et un âge moyen de 43 ans [1–4]. Deux théories convergentes sont avancées pour expliquer cette maladie [4, 5]: la théorie obstructive: les MA sont dues à une accumulation de mucus en amont d'une sténose de la lumière appendiculaire (adénocarcinome caecale, tumeur de l'appendice, tuberculose). La théorie néoplasique: la tumeur est responsable d'une hypersécrétion de mucus dans la lumière appendiculaire. Depuis les travaux de Woodruff et Mac Donald en 1940 plusieurs classifications histopathologiques ont été proposées. Celle de Wackym et Gray actuellement retenue divise les MA en 3 groupes: -les MA rétentionnelles non néoplasiques qui correspondent à une simple hyperplasie de la muqueuse arrangé en structure papillaire sans atypie, ni mitose. Dans ce cas l'appendice est macroscopiquement normal ou discrètement dilaté. -Les cystadénomes mucineux: l'appendice est dilaté et la lumière est tapissée par un épithélium mucosecretant uni stratifié avec des cellules cuboïdes ou en colonnes. Des formations papillaires, des atypies peuvent exister mais restent limitées à la muqueuse. -Les cystadénocarcinomes mucineux: haut degré d'atypie et de mitose, avec un EMI fréquent. Cliniquement la MA est asymptomatique dans 11 à 47% des cas. Ce caractère dépend du type histologique, en effet 80% des MA rétentionnelles et 30 à 50% des cystadénomes sont de découverte sur pièce opératoire de façon fortuite alors que les cystadénocarcinomes rarement asymptomatiques [6–9]. Les symptômes les plus fréquemment retrouvés sont une douleur de la fosse iliaque droite (présentes dans 27 à 64% des MA et dans 40 à 77% des cystadénocarcinomes), une masse abdominale (9 à 46% des cas), une OIA et une augmentation du volume d'une hernie ombilicale ou inguinale. Dans notre série tous les malades étaient symptomatiques et la douleur était présente dans tous les cas. Une complication à type de perforation, péritonite, volvulus appendiculaire, rectorragie, méléna ou une complication urologique peut aussi révéler une MA. La rupture se fait le plus souvent dans le péritoine et rarement dans le rétro péritoine. Les investigations para cliniques peuvent être de grand apport pour le diagnostic [3, 4, 6]. En effet sur un cliché de l'abdomen sans préparation des calcifications annulaires ou arciformes, avec un appendice porcelaine peuvent parfois exister.

L'imagerie basée sur le couple échographie -TDM est d'un intérêt capital pour le diagnostic préopératoire. L’échographie montre alors une masse kystique de la fosse iliaque droite indépendante des viscères abdominaux oblongue ou piriforme à grand axe vertical, aux contours nets venant au contact de la barrière gazeuse du cæcum [10]. La TDM constitue un examen capital et permet d'objectiver le raccordement entre la tumeur et le cæcum. La tumeur est souvent arrondie, bien limitée et parfois cloisonnée. La densité du contenu peut varier de celle d'un liquide aqueux à celle des tissus mous (de 10 à 45 UH) sans rehaussement après injection d'iode alors que la paroi se rehausse finement et de façon homogène. En cas de maladie gélatineuse, la substance mucoïde est de densité homogène ou hétérogène, liquidienne ou parfois de densité élevée. Elle peut être responsable de compression extrinsèque notamment sur la surface hépatique réalisant un aspect de « scalopping » très évocateur d'ascite gélatineuse. En présence d'une masse ou d'un épanchement d’étiologie inconnue, la laparoscopie est une phase fondamentale dans le diagnostic [10]. Un cystadénome ou à un cystadénocarcinome mucineux de l'ovaire associé est souvent de même type histologique que la mucocèle appendiculaire ou serait une localisation métastatique synchrone de la tumeur primitive appendiculaire [11]. Tous les auteurs [1–5] s'accordent pour recommander la seule appendicectomie en l'absence de signes histologiques de malignité et en présence d'une MA rétentionelle. En présence d'un cystadénome, une résection caecale est parfois nécessaire pour l'exérèse complète de la lésion. En cas de lésion maligne, l'hémicolectomie est considérée comme le traitement de choix et s'impose d'emblée en cas d'examen extemporané ou secondairement après le diagnostic histologique définitif certain, d'où l'intérêt de la réalisation systématique d'un examen anatomo-pathologique de toute pièce d'appendicectomie [3, 4, 6, 10, 11]. La voie d'abord coelioscopique doit être évitée étant donné les risques de récidive et de dissémination per-opératoire [8]. Les MA et les cystadénomes sont guéris par la seule chirurgie et ne récidivent pas après exérèse complète. Leurs taux de survie à 5 ans atteint 100%. Au cours du cystadénocarcinome les récidives sont fréquentes et le traitement adjuvant par radio-chimiothérapie n'est pas efficace et n'améliore pas la survie [7, 9]. De ce fait le cystadénocarcinome mucineux a un pronostic péjoratif surtout s'il est associé à un EMI métastatique. Sa survie à 5 ans ne dépasse pas 30% [10, 11].

## Conclusion

Les tumeurs mucosécretantes de l'appendice sont rares. Elles doivent être évoquées devant un syndrome appendiculaire atypique ou en cas de masse de la fosse iliaque droite. La découverte ou la persistance d'une masse de la fosse iliaque droite doit faire évoquer entre autres diagnostics celui de M.A et imposer la réalisation d'une échographie et/ou d'un scanner dont une meilleure connaissance des signes doit permettre un diagnostic précoce. Lorsqu'elles sont diagnostiquées et traitées, elles sont de pronostic favorable. La hantise doit être toujours d’éliminer un cystadénocarcinome mucineux de pronostic différent et de traitement chirurgical lourd.
